# Effectiveness of a Mindfulness-Based Intervention Program to Improve Communication and Stress Coping Skills in University Students

**DOI:** 10.3390/ejihpe14070128

**Published:** 2024-06-29

**Authors:** Ana María Morales-Rodríguez, Francisco Manuel Morales-Rodríguez

**Affiliations:** 1 Department of Journalism, Faculty of Communication Sciences, University of Málaga, 29010 Malaga, Spain; amoralesr@uma.es; 2 Department of Educational and Developmental Psychology, Faculty of Psychology, University of Granada, 18011 Granada, Spain

**Keywords:** mindfulness, communication, intervention

## Abstract

Investigating the contribution of mindfulness training to psychological well-being and quality of life in the university setting is of interest. The objective of the study is to present a comparative analysis of the scores in the variables of self-efficacy, resilience, coping strategies, and communication skills before and after the application of an intervention program based on mindfulness. An ex post facto cross-sectional design and a convenience sample of participants were adopted. The participants were students belonging to Education Sciences who benefited from the activities of the program. Instruments were administered to assess mindfulness, self-efficacy, resilience, coping strategies, and communication skills. The correlations of the mindfulness variable with the other psychoeducational variables evaluated were also analyzed. The results indicate an increase in the scores in the selected variables of mindfulness, resilience, communication skills, and some of the coping strategies considered productive or functional such as problem solving, self-criticism, emotional expression, desiderative thinking, social support, and cognitive restructuring. Statistically significant correlations were also observed between the variable mindfulness and those of perceived self-efficacy, resilience, coping strategies, and communication skills. The development of mindfulness training programs in the university setting is necessary to contribute to the improvement of more adaptive coping skills and the promotion of resilience.

## 1. Introduction

Research evaluating coping strategies for daily stress, self- and life-acceptance and other personal competencies, perceptions, self-appraisals, feelings of perceived self-efficacy and communication skills in the university setting has been rather scarce. Likewise, there have been few studies assessing the effects of mindfulness in university students, despite the existing evidence of its benefits in primary and secondary education. There is an extensive review of studies that have researched the variables under study, which are described in the following paragraphs, but very few have focused on the university context with a Spanish population and analyzed the effects of a mindfulness-based intervention program, as is performed in this study. In this sense, in our context there are some studies that have developed an intervention program focused on mindfulness and emotional regulation for professionals [1,2], those aimed at secondary school students [3], or those focused on examining only the relationships between some of these variables in a non-Spanish university population [4,5]. However, there are hardly any studies focusing on a psychoeducational intervention program with a sample of university students in Spain. This type of program was considered very necessary within our context, where we are aware that for first-year students, the university environment is one of the most academic and daily stress-inducing contexts [6,7]. In general, in the university context there is still a need to promote activities for the development of mindfulness-based emotional awareness, and the promotion of social and communicative skills that help to cope with stress and adversity and allow for better well-being and a more positive classroom environment.

An author [8] defines full consciousness in the following way:

Full consciousness is the quality of awareness that emerges when we deliberately concentrate the mind on the present moment. Attention is directed to the experience that is lived and suffered, without any filter (it is accepted as it is), without formulating judgment (good or bad, desirable or not), without expecting anything in particular. (p. 70).

Similarly, another study [9] (p. 34) defines it as “the practice of intentionally focusing attention on the present moment without making value judgments”, which includes two components of the process of perception and emotional regulation change: (a) self-regulation of attention in the present moment, and (b) minimizing value judgments. Along the same lines, mindfulness has been considered a protective factor against academic stress, which authors such as Brown and Ryan [10] (p. 822) understand as a “state of being attentive to and aware of what is taking place in the present”.

It is therefore “a process of attentional self-regulation that allows the development of present moment awareness” [11]. According to a previous study [12], it involves definitions that can be unclear, such as:

“Mindfulness is an immediate, non-valuative and uninterrupted awareness that registers physical, emotional and mental processes without continuity between instants” (Mazzola and Rustherholz, based on Grossman [13]) (p. 34)). Mindfulness is also defined as:

“Mindfulness is a powerful tool with which to achieve deep awareness, serenity and concentration. It promotes self-awareness, self-empathy, self-acceptance, self-directed behavior, and self-help” [14].

This article assumes a psychoeducational theoretical model related to the importance of contributing with research in a university setting to the development of the affective dimension of transversal competencies and the need to carry out more activities and training programs in such settings [15,16] considering variables such as communication skills, effective coping, mindfulness and students’ resilience capacity. This should be in line with the theoretical model proposed by other authors [2,4], who argued that for the achievement of positive or effective coping skills, a central component of mindfulness known as the “theory of reperceiving” is fundamental. This component emphasizes emotional and behavioral flexibility in addition to other cognitive aspects. However, as shown in a recent investigation [17], the same coping strategies may be considered adaptive or functional in certain situations and circumstances and maladaptive or dysfunctional in others.

According to several studies [18,19,20], resilience, coping strategies and social and communicative skills are the variables considered relevant in the educational field when examining or investigating the effects of the dependent variable mindfulness.

Concerning resilience, a previous study [18] used a sample of university students and found that ego resilience buffered the positive correlations between academic stress and anxiety and showed inverse correlations with the variable mindfulness. In this sense, resilience implies a predisposition to reduce anxiety [21] and allows for more effective coping with stressful situations in students [22]. In the same line, another study [23] found positive correlations between mindfulness and resilience in a sample of African American college students and highlighted the need to promote mindfulness skills to help develop non-reactivity and resilience to stress among students. Similarly, a previous study [24] showed that resilience is one of the main mediating variables of the positive impact of mindfulness on subjective well-being.

Other research [25] also evaluated the impact of mindfulness on variables such as resilience, mental well-being and self-perceived daily stress in a sample of university students in social work, finding statistically significant differences in resilience, stress and well-being post-intervention scores in the treatment group compared to the non-participating control group. Specifically, the resilience scores increased, well-being improved, and anxiety and stress levels decreased after student participation in a program for enhancing mindfulness. Likewise, from a qualitative perspective, the open-ended questions answered by the students also showed an improvement in coping strategies and resilience. Regarding self-efficacy, research [26] found correlations between mindfulness, stress and self-efficacy in a sample of university students.

Coping is “changing thoughts and acts an individual uses to manage the external and/or internal demands of a specific person-environment transaction that is appraised as stressful” [27] (p. 34). Regarding the relationships between mindfulness, socioemotional skills and coping strategies, research [28] found statistically significant correlations between mindfulness and positive and negative affect in a sample of 125 university students who participated in a mindfulness training program; the authors highlighted the importance of these relationships for an adaptive emotional response in managing daily life dynamics. In the same line, Moscoso [11] indicated that the practice of mindfulness can contribute to the improvement of socioemotional skills. Another study [4] found statistically significant positive correlations between mindfulness and the so-called rational coping in a sample of 135 first-year university students; inverse correlations were also found for mindfulness with stress and emotional and avoidant coping. The same study found statistically significant positive correlations of mindfulness with an adaptive coping style and statistically significant inverse correlations between mindfulness and maladaptive coping styles. Other research [29] points out the benefits of mindfulness for coping with stress experienced by university students in different academic, personal and social situations such as those related to study or freshmen having to live alone, separated from their families for the first time. Other research [30] found in a sample of 157 undergraduate students, mostly women, that greater employment of mindful emotion-focused coping strategies—such as acceptance and non-blame—mediate the effects of mindfulness on positive and negative affect. They indicated that these strategies do not replace but complement problem-focused coping and that it is necessary to promote health education that includes mindfulness programs or interventions for students. 

Therefore, the objectives of this study were: (1) to analyze the effects of a mindfulness and emotional education intervention on the selected variables of self-efficacy, resilience, coping strategies and communication skills, by comparing data collected at the beginning of the program with post-intervention data; (2) to evaluate the degree of satisfaction that the activities brought to the participants; and (3) to examine the relationships between mindfulness and perceived self-efficacy, personal competence and self-acceptance, coping strategies and communication skills. It was hypothesized that: (1) Significant differences are expected to be found in communication and mindfulness skills, self-efficacy, resilience and coping strategies before and after participation in the intervention program; (2) Mindfulness levels are related to levels of self-efficacy, resilience, coping strategies considered *a priori* as productive, and communication skills.

## 2. Materials and Methods

### 2.1. Design and Procedure

The study was approved by the corresponding University Ethics Committee. Informed consent was obtained, anonymity and confidentiality of the data obtained were guaranteed, and the study could be abandoned at any time. The study had an ex post facto design. The questionnaires selected to evaluate the effects of the program were administered collectively in the classroom before the start of the intervention program and after its completion in a control group (without intervention) and in an experimental group (before the program’s intervention) with very similar sociodemographic features and starting levels such as the same age, year group, degree, training and initial level of knowledge and expertise in these types of activities. The participants were asked directly before participating in the program about their level of knowledge about mindfulness and if they had previous experience in its practice, verifying that their level was the same and that none of them had previous experience in the practice of mindfulness. Participants also reported that they did not have anxiety disorders or issues when they were asked about it. The program was held during the academic year 2021/2022 for a duration of four months with two-hour seminars per week in which small groups discussed different activities based on the importance of mindfulness-based emotional education in a face-to-face and non-face-to-face manner. The data were collected between September 2021 and July 2022. Two phases are identified. In the first phase, (pre-test phase), instruments for the assessment of mindfulness, self-efficacy, resilience, coping strategies and interpersonal skills were administered. In the second phase (post-test intervention), the mindfulness-based intervention program was developed and instruments for the assessment of mindfulness, self-efficacy, resilience, coping strategies and interpersonal skills were administered again after the program was fully developed. In this second phase four sessions were developed in which face-to-face and non-face-to-face activities were planned during the small group seminars of the classes, with a minimum of two face-to-face hours per week. The following activities were carried out by participants during the first session (6 h), with some of the tasks being face-to-face and others non-face-to-face: (a) listening to audio files and watching videos on mindfulness. During the second session (9 h), some activities of Giménez-Dasi’s intervention program [31] were adapted, such as the ones related to being mindful of the emotions with mindfulness, performing mindfulness activities and tests focusing on the breathing, and the strategy of cognitive restructuring applied to socio-emotional situations. In the third session (9 h), discussion points and activities were developed, adapted from the second part of Baró’s [32] manual on how to communicate, how to deal with criticism in a positive way and how to adopt positive language; and especially from another book [33] related to the ability of urge surfing, the existence of unrealistic expectations, the so-called fusion with unhelpful thoughts, coping with unpleasant emotions and the degree of detachment from one’s values and goals. In the fourth session (8 h) activities adapted from Mazzola and Rusterholz’s book [12] on the integration of mindfulness for coping with everyday stress were carried out together with activities for ‘Managing stressful everyday situations’, ‘Strengthening mindfulness (mindful breathing, feet on the ground and travelling through the body’ and ‘assessing the facets of mindfulness in everyday life’).

### 2.2. Participants

The participants were 77 university students, most of whom were women (68%), that followed studies in the Primary Education Undergraduate Degree of the Faculty of Education Sciences. Their ages ranged between 18 to 22 years old. A control group comprised 49 students (69% of whom were women) of another cohort within the Primary Education Undergraduate Degree who did not participate in the activities and tasks of the intervention program. The two groups (experimental and control) are comparable by sex and age as it is another group of students of the same degree and academic year.

The following inclusion criteria were considered: (1) age between 18 and 24 years; (2) not being a part-time student. Data from participants who did not complete any of the questionnaires before or after the intervention program were deleted. Other selection criteria that were taken into account to avoid bias in the interpretation of the results were that participants were not familiar with mindfulness or they were not practicing it, did not have mood disorders such as anxiety, depression or other underlying conditions, and were not attending the psychology clinic service for students at the University for other reasons.

### 2.3. Instruments

We administered the following instruments to evaluate the effects of the program.

The Mindfulness assessment [8] consists of 15 items rated on a six-point Likert-type scale where 1 = almost always, 2 = very often, 3 = often, 4 = a little frequent, 5 = rarely and 6 = never. The scores are summed and the result is divided by 9. The higher the score, the greater the predisposition to mindfulness. The internal consistency for this sample was 0.89. This instrument shows adequate evidence of validity with previous work in Spanish [34,35].

The General Self-Efficacy Scale [36], validated in Spanish [37]. This is a ten-item instrument with responses ranging from 1 to 10, where 1 is “totally disagree” and 10 is “totally agree”. It measures the stable feeling of personal competence to effectively handle a wide variety of stressful situations; the higher the score, the higher the general self-efficacy. This scale has adequate psychometric properties (α = 0.88).

The Resilience Scale adapted in Spanish [38] (Spanish version of the Resilience Scale by Wagnild) [39]. This scale comprises 14 items and allows the assessment of two factors: (a) personal competence and (b) acceptance of oneself and life. Its response format is a seven-point Likert-type scale where 1 is “Strongly disagree” and 7 is “Strongly agree”. Higher scores reflect higher levels of resilience. Its internal consistency is adequate (α = 0.80).

The Coping Strategies Inventory [40] adapted in Spanish [41]. This instrument has an open-ended question where respondents should think about a situation that worries them and a second part with 40 items to be rated on a five-point Likert-type scale (0 = “not at all” and 4 = “totally agree”). These items are grouped into different subscales: problem solving, self-criticism, emotional expression, desiderative thinking, cognitive restructuring, problem avoidance and social withdrawal. The internal consistency for the sample in this study ranged from 0.75 to 0.89.

An Interpersonal Skills Scale by Torbay et al. [42]. It consists of 20 items rated on a five-point Likert-type scale where 1 is “never”, 2 “very seldom”, 3 “sometimes”, 4 “often” and 5 “always”. The higher the total score, the better social skills are evidenced. The instrument presents adequate psychometric properties (α = 0.91) and adequately valid indices [43]. Validity in Spanish was verified using the interjudge agreement method in a previous study [43].

A continuous evaluation instrument to assess the degree of activity-induced satisfaction during the development of the program following a four-point scale: 1 = not at all satisfied; 2 = partly satisfied; 3 = quite satisfied and 4 = very satisfied. This continuous evaluation instrument for assessing satisfaction is comprised of a single question. It was administered after completing the development of the program.

Mindfulness-based intervention program:

An intervention program based on mindfulness and emotional education. It has been described in the design and procedure section.

### 2.4. Data Analysis

The statistical analysis was performed using SPSS Windows software version 23 (computerized statistical package). Levene’s test was used to determine whether there was equality of variances and the assumptions of homoscedasticity, normality and independence of residuals were also verified by parametric analyses and, consequently, the choice of statistical tests used for data analysis.

The descriptive statistics of the sample were analyzed (percentages, means, standard deviations). Student’s *t*-test for related samples was applied to see if there were statistically significant differences in the evaluation of mindfulness, perceived self-efficacy, personal competence and self-acceptance, coping strategies and communication skills. A Student’s *t*-test was carried out to compare the means of the study variables in terms of sex to determine whether there were significant differences between the two groups.

The analysis of the relationships between these variables was carried out using Pearson’s correlation analysis, which was tested beforehand for non-collinearity and compliance with the assumptions of normality. A *p*-value lower than 0.05 was considered statistically significant in all analyses.

## 3. Results

Table 1 presents the scores on the control program comparing the scores before and after on mindfulness, self-efficacy, resilience, coping strategies and communication skills (a different group that did not take part in the program).

Table 2 presents the effects of the mindfulness-based intervention program comparing the scores on mindfulness, self-efficacy, resilience, coping strategies and communication skills before and after participation in the program (experimental group). An increase was found in the mindfulness scores, in the self- and life-acceptance factor of the resilience variable, in the productive or functional coping strategies of problem solving, self-criticism, emotional expression, desiderative thinking, social support, cognitive restructuring, as well as in the communication skills variable selected for evaluation. A decrease in the unproductive coping strategy called social withdrawal was also found.

The participating sample indicated that the program activities generated a high degree of satisfaction (a mean score of 3.76 and a standard deviation of 0.43 according to responses on a Likert-type scale where 1 = “not at all satisfactory” and 4 = “very satisfactory”) about their usefulness in the academic and personal spheres.

Table 3 shows the correlations referring to data collected at the beginning of the intervention between mindfulness, perceived self-efficacy, personal competence and self-acceptance, coping strategies and communication skills in university students. The results showed positive correlations between mindfulness and the total score for perceived self-efficacy, resilience, coping strategies and communication skills.

Table 4 below shows the analysis of mean differences in the study variables according to sex. Females showed higher scores in the coping strategy of emotional expression compared to males.

## 4. Discussion

The general objective of this study was to evaluate the effects of an intervention program based on mindfulness of emotions on certain psychoeducational variables in the university setting, while examining the relationships between them in a sample of students. The results obtained showed significant changes in the selected variables after the completion of the mindfulness program. The activities conducted related to mindfulness-based emotional awareness and coping strategies used in different situations, attention to breathing and the promotion of an effective communication style contribute, in that regard, towards the acquisition and development of transversal socio-emotional skills that lead to the improvement of resilience and a more functional, adaptive or effective coping. This is consistent with previous studies that show that the design of this type of action or intervention to improve self-regulation and emotional management that facilitates active listening and communication skills contribute to optimizing the teaching/learning process with the use of a more diverse teaching methodology in the university context [44]. These interventions are focused not only on building knowledge but also on a humanistic-interpersonal dimension for the construction of the person [45] which have an impact on the influence of changes in this type of variables such as those assessed here. Along the same lines, another study [46] highlights the importance of mindfulness to try to optimize health and well-being in young Spaniards. Moreover, following the impact of the COVID-19 pandemic situation in the field of education [7], students and young people have been demanding this type of training related to emotional education to an even greater extent.

Scores improved in variables such as resilience (acceptance of oneself and life) congruent with previous research [18] that found relationships between ego resilience and mindfulness in a sample of university students; both variables also correlated negatively with the emotional dimension in its negative aspect, specifically with the levels of anxiety and depression. This result is also consistent with previous research where the relationship between the variables mindfulness and resilience is analyzed, having found a positive and statistically significant correlation between said variables [24]. Along the same lines, another study [47], although in a different context, found that training that generates changes in mindfulness is also associated with changes in variables such as resilience, which, in turn, can lead to better management of emotions and better coping with emotional and behavioral problems during youth and adolescence. Other research shows the links or benefits of mindfulness with regard to the impact of mindfulness on the resilience variable [48]. These benefits can in turn contribute towards improving the teaching and learning process by increasing the students’ motivation and sense of achievement [49]. Similarly, other research [23] found in a sample of African-American university students, mostly women with an average age of 20.66 years, that the variable mindfulness (a variable related to being able to pause before responding to the stimulus) correlated with the variable resilience.

This program also improved scores in the use of coping strategies for stressful situations considered adaptive or functional as well as in the acquisition and development of effective communication skills. Likewise, the use of coping strategies considered at first unproductive or maladaptive, such as social withdrawal, decreased. These results are in line with previous research indicating that mindfulness intervention is associated with less maladjustment and an increase in adaptive social interaction behaviors [3,50] that may include effective social and communication skills. Likewise, gratitude-building activities that are transversally included in this type of program can contribute to a decrease in aggressive behaviors and thus, violence prevention [51].

The previous scientific literature has shown that mindfulness programs can be effective in reducing negative emotional states [11,52], which is fundamental for the improvement of social and communicative skills. Another previous study [28] posited that mindfulness can promote affective adaptive experiential behaviors and mindfulness was associated with levels of negative affect. This can be considered supportive of our study that found associations between levels of mindfulness and emotional dimensions such as emotional expression.

It is worth noting the existence of statistically significant correlations between mindfulness and resilience, also evidenced in some previous studies [18,23]. A previous study found correlations between ego resilience and social functioning [53]. Another study [40] also showed positive associations between mindfulness and effective or functional coping styles. In the same line, it has been found that mindfulness programs have benefits for the development of resilience and more effective coping strategies among students [25,54]. Mindfulness is thus a relevant psychological variable to be considered in the design of psychoeducational intervention programs for developing socioemotional competencies such as those related to coping with stress and adversity. Other researchers [23] highlight the need to develop more programs and interventions to improve mindfulness in the university context.

Future studies could deepen the relationships between the variable mindfulness and academic achievement in university students in this context. In another cultural context [55], students with higher academic achievement exhibited greater mindfulness. However, the relationships between these variables of mindfulness and academic achievement were weak, and explained little variance of the academic achievement variable [55].

Regarding the degree of satisfaction, the participants showed a high degree of involvement and satisfaction with activities in the program. They considered them to be useful for coping with adverse situations in both their academic and personal lives. This result is to be expected considering the voluntary nature of participation both in the activities to be carried out in the classroom and in other activities proposed to be practiced at home. In this sense, as shown in a previous study [25], it is important that participation in this type of training activities focused on mindfulness be voluntary and not compulsory for students in order to improve their self-care and well-being.

### Limitations of the Study and Future Line of Research

Limitations of this study are related to the subjectivity of self-reported measures and its cross-sectional design. With regard to the level of generalization this study achieves, it should be noted that the sample of participants that took part in this study follow studies in the Degree in Primary Education of the Faculty of Education Sciences, and we are aware that there might be differences between the profile of these students and those of other degrees within the Educational Sciences, Psychology and other fields belonging to different areas of knowledge. It should also be noted with regard to the factors that may have influenced these results, that the proportion of female participants is higher than that of male participants, and it would therefore be necessary to further investigate the role that the variable sex could play in the generation of possible differences in the assessed variables. It should also be pointed out that the participants were taking subjects such as educational psychology, which, by including in its content topics such as education and emotional management, social competence, social interaction, etc., has allowed the students to receive continuous feedback and knowledge related to the variables analyzed in the program. Therefore, in another degree they could have possibly worked with fewer activities, or to a lesser extent, than with those suggested in this program.

Future research could delve more specifically into the effects of mindfulness-based programs on students’ internalized symptomatology (such as academic anxiety), as well as on their life satisfaction, psychological well-being and the social and educational climate in the classroom. Future studies could also apply other types of analysis, such as those based on artificial intelligence and the predictive capacity of neural network models, to verify the relationships between the selected constructs.

## 5. Conclusions

This study found statistically significant differences before and after applying a mindfulness-based intervention program to improve socioemotional and communication skills. Improvements were recorded in self- and life-acceptance, which are aspects of resilience, functional or productive coping strategies such as problem solving, desiderative thinking, adequate emotional expression, social support and social and communication skills. Statistically significant correlations were also found between mindfulness and perceived self-efficacy, personal competence, self- and life-acceptance (resilience), and functional coping strategies. Statistically significant positive correlations were identified between mindfulness and communication skills in the students participating in this study.

This type of program is necessary in university classrooms to improve the resilience, coping and emotional management capacity of university students whose academic stress has been accentuated following the COVID-19 pandemic. The insights obtained can support the improvement of counseling services for university students, especially first-year students, through mindfulness-based activities, courses and programs.

## Figures and Tables

**Table 1:** Comparison of pre-post means of the study variables in the control group of university students.

Variables	Pre		Post		Student’s *t*-Test		
	M	DT	M	DT	t	gl	*p*
Mindfulness	19.71	26.97	28.19	29.03	−1.50	40	0.14
Self-efficacy	53.05	33.53	48.32	40.21	0.73	40	0.47
Resilience							
Personal competence	52.04	7.60	60.91	3.91	1.53	40	0.37
Self- and life-acceptance	14.91	3.00	15.61	1.06	−2.33	40	0.26
Coping strategies							
Troubleshooting	12.76	5.95	11.86	6.15	1.33	42	0.19
Self-criticism	8.24	6.25	8.26	5.63	−0.03	40	0.97
Emotional expression	7.82	5.18	7.47	5.43	0.59	40	0.56
Desiderative thinking	8.87	5.19	8.39	5.65	0.82	40	0.41
Social withdrawal	6.11	4.81	7.13	5.39	−1.53	40	0.13
Problem avoidance	6.00	3.81	7.11	4.56	−1.98	40	0.55
Cognitive restructuring	10.38	5.15	11.23	6.17	−1.09	40	0.28
Social support	8.48	3.33	8.90	4.01	−5.25	40	0.12
Communication skills	63.10	32.10	60.28	38.06	0.91	49	0.37

gl: degrees of freedom; *p*: significance level; t: Student’s *t-statistic* for dependent samples; M: mean.

**Table 2:** Comparison of pre-post means of the study variables in the experimental group of university students participating in the mindfulness program.

Variables	Pre		Post		Student’s *t*-Test		
	M	DT	M	DT	t	gl	*p*
Mindfulness	38.00	27.23	57.15	9.93	−3.17	75	0.00
Self-efficacy	60.70	31.42	54.80	36.14	1.32	74	0.19
Resilience							
Personal competence	61.11	1.18	62.83	3.92	−1.02	75	0.35
Self- and life-acceptance	16.73	1.41	21.50	1.04	−12.42	75	0.00
Coping strategies							
Troubleshooting	8.62	6.40	13.12	4.39	2.41	75	0.03
Self-criticism	5.29	4.25	9.23	4.67	2.47	75	0.02
Emotional expression	8.06	7.02	11.26	3.86	−24.35	75	0.00
Desiderative thinking	8.94	7.06	14.16	3.60	−25.68	75	0.00
Social withdrawal	10.33	1.74	7.11	5.78	2.12	75	0.04
Problem avoidance	7.50	6.29	6.62	3.91	−0.46	75	0.65
Cognitive restructuring	8.11	6.27	11.67	4.82	2.04	75	0.05
Social support	8.46	7.33	14.47	5.04	3.10	75	0.00
Communication skills	58.40	38.55	68.40	32.27	2.12	75	0.03

gl: degrees of freedom; *p*: significance level; t: Student’s *t-statistic* for dependent samples; M: mean.

**Table 3:** Correlations between mindfulness, perceived self-efficacy, personal competence, self-acceptance, coping strategies, and communication skills in university students.

Variables	1	2	3	4	5	6	7	8	9	10	11	12	13
1		0.41 **	0.51 **	0.32 *	0.33 *	0.33 *	0.41 **	0.16	0.34 **	0.27 *	0.33 *	0.32 *	0.50 **
2			0.71 **	0.64 **	0.48 **	0.21	0.24	−0.02	0.39 **	0.45 **	0.20	0.28 *	0.44 **
3				0.78 **	0.51 **	0.10	0.25	0.10	0.46 **	0.34 **	0.05	0.34 **	0.70 **
4					0.54 **	0.46 **	0.12	0.49 **	0.55 **	0.31 **	0.01	0.42 **	0.54 **
5						0.20	0.27 *	0.07	0.62 **	0.13	0.20	0.37 **	0.53 **
6							0.25	0.51 **	0.29 *	0.34 *	0.34 *	0.21	0.27 *
7								0.39 **	0.58 **	0.33 *	0.05	0.66 **	0.42 **
8									0.25	0.21	0.18	0.28 *	0.16
9										0.35 *	0.20	0.69 **	0.46 **
10											0.47 **	−0.03	0.15
11												−0.03	−0.10
12													0.41 **
13													

* *p* < 0.05; ** *p* < 0.01; 1. Self-perceived self-efficacy; 2. Personal competence (resilience); 3. Self- and life-acceptance (resilience); 4. Self-acceptance of self and life (resilience); 5. Problem Solving (coping strategy); 6. Self-criticism (coping strategy); 7. Emotional expression (coping strategy); 8. Desiderative thinking (coping strategy); 9. Social withdrawal (coping strategy); 10. Problem avoidance (coping strategy), 11. Cognitive restructuring (coping strategy); 12. Social support (coping strategy); 13. Communication skills.

**Table 4:** Comparison of means (Student’s *t*-test) to determine differences by sex for the study variables.

Variables	Men		Women		Student’s *t*-Test		
	M	DT	M	DT	t	gl	*p*
Mindfulness	26.00	26.30	35.20	29.49	−0.77	75	0.45
Self-efficacy	58.76	35.65	61.56	30.62	−0.30	74	0.76
Resilience							
Personal competence	62.11	9.51	62.23	7.10	−0.05	75	0.95
Self- and life-acceptance	15.17	3.83	14.56	2.77	0.72	75	0.47
Coping strategies							
Troubleshooting	11.52	6.34	13.90	4.85	−1.39	75	0.17
Self-criticism	8.86	5.23	8.50	5.52	0.22	75	0.82
Emotional expression	5.85	4.51	10.45	4.95	−3.14	75	0.00
Desiderative thinking	8.42	5.92	9.86	5.23	−0.85	75	0.39
Social withdrawal	8.23	5.64	6.39	4.25	1.22	75	0.23
Problem avoidance	7.36	4.54	7.38	4.93	−0.01	75	0.98
Social support	6.33	5.31	6.84	3.30	−0.39	75	0.69
Communication skills	60.07	35.31	69.62	34.80	−0.36	75	0.34

gl: degrees of freedom; *p*: significance level; t: Student’s *t-statistic* for dependent samples; M: mean.

## Data Availability

The data could be requested by the scientific community under the ethical terms to be determined.
